# Case for Thought: Meckel's Diverticulum in the Adult Population

**DOI:** 10.7759/cureus.24494

**Published:** 2022-04-26

**Authors:** Melanie N Rayan, Xiaolan Tang, Maher Khazem, William Clements, Christopher Bray

**Affiliations:** 1 Internal Medicine, University of Central Florida College of Medicine, Graduate Medical Education/HCA Florida North Florida Hospital, Gainesville, USA

**Keywords:** meckel's diverticulum, gastrointestinal hemorrhage, gastrointestinal bleeding, gastrointestinal tract complications, vitelline duct remnant, meckel's diverticulum in adults, incidental meckel's diverticulum

## Abstract

Meckel’s diverticulum (MD) is a well-known gastrointestinal (GI) congenital anomaly that is generally considered a disorder in neonates or a “clinically silent” disorder in adults. While prevalent in children, MD is not often considered as a differential in the etiology of GI bleeding in the adult population. We describe a case of MD presenting as a copious GI bleed in a 65-year-old male, which was found after numerous diagnostic studies. Our case report aims to challenge the idea that Meckel’s diverticulum is solely a neonatal disorder, identify this vitelline duct remnant as a potential cause of GI pathology in the adult population, and discuss the detection and management of this congenital abnormality when found in the older population.

## Introduction

In a typical developing embryo, the primitive midgut receives nutrients from the mother’s uterine yolk sac via the yolk stalk or vitelline duct, a structure formed as an outpouching of the embryo’s bowel wall [1]. This embryologic “nutrient conduit” routinely forms around the fourth week of gestation and involutes or resorbs by around the fifth to ninth week [1]. When the vitelline duct persists or fails to involute completely, it is termed Meckel’s diverticulum (MD) and considered a congenital abnormality. MD is the most prevalent congenital abnormality of the gastrointestinal (GI) tract, reportedly present in between 0.3% and 2.9% of the general population [2]. This persistent outpouching of the primitive gut can lead to an array of clinical problems, which include GI bleeding, volvulus, intussusception, the passage of meconium through the umbilicus, or obstruction, and is conventionally taught to present in neonates [2]. We present an uncommon case of a 65-year-old male with a lower GI bleed of unknown origin that presented secondary to complications of a previously clinically silent MD. Following an array of diagnostic studies including two colonoscopies, an endoscopy, a computed tomography angiography (CTA), a tagged red blood cell (RBC) scan, and selective mesenteric angiography, an exploratory laparotomy eventually revealed this unanticipated etiology. While hemorrhoids, diverticulitis, and angiodysplasia should certainly be higher on the differential, MD should be considered in adults in whom studies are inconclusive in identifying the source of a GI bleed.

## Case presentation

Our patient was a 65-year-old male who presented to our emergency department (ED) for a syncopal episode and a two-day history of rectal bleeding. His past medical history (PMH) was notable for hypertension, type II diabetes mellitus, and coronary artery disease. The patient endorsed multiple episodes of abdominal discomfort and tenesmus followed by bright red rectal bleeding for a duration of two days. The patient’s rectal bleeding was unaccompanied by stool burden, and his last bowel movement preceded the onset of bleeding. His symptoms culminated in a syncopal episode with loss of consciousness and associated head trauma, prompting his ED visit. He complained of constipation with an increase in flatulence for six to eight months, with bowel movements occurring only twice weekly. He observed his stools to be hard and difficult to pass. He denied a history of diverticulosis, hemorrhoids, and rectal bleeding in the past. A review of systems was positive for tactile fevers of one-week duration, loss of appetite, poor oral intake, and an unintentional weight loss of 50-60 pounds in the past six months. His last screening colonoscopy was 10 years prior, during which multiple benign polyps were resected. The patient was advised to follow up within five years, however, he had not been compliant with his follow-up requirement. Family history was notable for Hodgkin's lymphoma in his father. His social history was positive for prior use of cocaine and tobacco. He smoked two packs of cigarettes daily for 35 years; however, he quit four years prior to presentation. He denied any current or former alcohol use.

Upon initial presentation to the ED, he was found to be in hypovolemic shock, which was responsive to fluid resuscitation. At the bedside, the patient appeared pale and anxious, with stable vital signs. His abdomen was soft and tender to palpation in the bilateral lower quadrants. Bowel sounds were present and normal. Rectal examination showed no obvious hemorrhoids. Thirty cc of bright red blood with clots was observed. Other aspects of his physical exam appeared normal. He stated that he had continued to have a few episodes of rectal bleeding while in the ED. Routine labs returned normal except for hemoglobin of 11.2 gm/dL, and the basic workup for syncope was negative. He was given a dose of pantoprazole in the ED, a type and screen was performed, and blood products were placed on standby.

The patient was admitted to the medical unit and consults were placed to gastroenterology (GE) and general surgery (GS). He was made Nil Per Os (NPO) and started on scheduled pantoprazole and an octreotide drip. His home aspirin and clopidogrel were held. Computed tomography (CT) of the abdomen and pelvis with GI protocol showed diverticula in the colon with no acute source of bleeding. On Day 2 of hospitalization, continued bleeding prompted GE to perform an endoscopy and colonoscopy. The endoscopy showed no abnormalities except for a small hiatal hernia. The colonoscopy was inadequate due to poor bowel preparation and the presence of gross maroon blood throughout the colon. Mild external and internal hemorrhoids were noted with diffuse diverticulosis in the entire colon. A nuclear medicine bleeding scan was subsequently performed, which showed a possible active bleed in the right upper quadrant, attributable to a diverticular source or a mass at the hepatic flexure. An abdominal aortogram and selective three-vessel arteriogram were performed, which showed no bleeding in the super mesenteric artery (SMA), inferior mesenteric artery (IMA), and celiac angiograms. During the first two days of hospitalization, the patient continued to have rectal bleeding with hemoglobin dropping to a low of 6.6 gm/dL. He required a total of four units of packed red blood cells (pRBCs) and one unit of platelets during this time. His octreotide drip and pantoprazole were discontinued due to low suspicion of an upper GI bleed. Due to continued bleeding, a repeat colonoscopy was performed on Day 3 of hospitalization, which appeared normal except for gross hematochezia throughout the colon. A large number of clots were present within the colon and terminal ileum, causing suspicion of a possible small bowel bleed. Due to the volume of bleeding and transfusion requirement, the decision was made for an exploratory laparotomy to be performed when the patient was hemodynamically stable, with possible colectomy if an active source of bleeding was identified.

**Figure 1:**
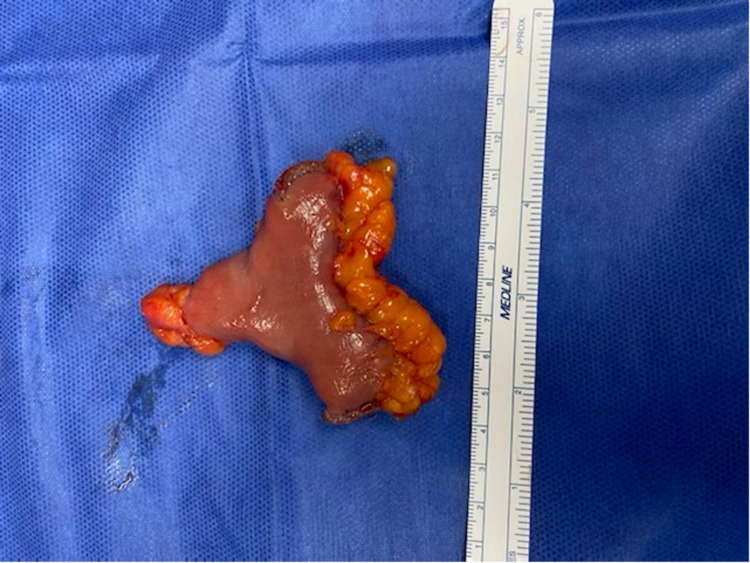
Gross image of resected Meckel's diverticulum

On Day 4 of hospitalization, the patient was started on a diet, which he tolerated well. On Day 5 of hospitalization, an exploratory laparotomy was performed. An MD with a wide base approximately 2 feet from the ileocecal valve was identified. Attempts were made to perform a push enteroscopy, however, continued coiling of the scope due to a redundant stomach prevented its advancement beyond the ligament of Treitz, hence no mucosal exam was possible. The Meckel’s diverticulum was resected (Figure 1) and end-to-end anastomosis was performed.

Postoperatively, the patient was kept NPO for two days, following which he was started on a clear liquid diet with a bowel regimen. He experienced a challenging postoperative course complicated by ileus, pneumonia, and an upper extremity deep venous thrombosis (DVT) leading to a small sub-segmental pulmonary embolus (PE). He was started on antibiotics promptly, however, full-dose anticoagulation treatment was delayed due to continued rectal bleeding. He required an additional four units of pRBCs during his postoperative course. One week following his surgery, the rectal bleeding began to slow down and normal bowel function returned. He was started on full-dose anticoagulation with unfractionated heparin for a few days, and upon tolerating this treatment well, he was transitioned to twice-daily dosing of low molecular weight heparin. He was discharged to a rehabilitation facility on postoperative Day 13 with follow-up appointments scheduled with hematology and general surgery.

## Discussion

MD is found in 0.14-4.5% of autopsy series, with a male/female ratio of 1.9 [3]. MD is most often asymptomatic in adults, discovered incidentally during surgeries for other purposes or through imaging as an incidental finding. However, we propose that in adult cases where initial diagnostic studies are unable to identify a source of a GI bleed, MD be considered as a potential etiology.

Bleeding from MD might be insidious or acute and massive. One study of radionuclide scanning reports sensitivity and a positive predictive value of 60% with a negative predictive value of 75% [4]. In adult patients, particularly in young adults <40 years of age with GI bleeding but no identifiable source with standard evaluation, which includes endoscopy/colonoscopy, CTA, small bowel studies, or radionuclide scanning, suspicion for MD as a source of lower gastrointestinal bleeding should be introduced, and diagnosis by means of exploratory laparotomy encouraged. The lifetime risk of complications related to MD is estimated at 4%, with complications seen more often in males than females (sex ratio = 2.8). Common complications of MD in adults include GI bleeding (8-63%) [5-6], obstruction (14-40%) [7-8], diverticular inflammation (58%) [9-10] and tumor (begin or malignant) [11-13].

Treatment of symptomatic MD is surgical resection. However, there is no clear recommendation regarding the incidental discovery of an MD in the adult population. Those who favor abstention argue that the postoperative complications that occur after prophylactic resection of MD are much higher than the risk of complications related to MD itself, therefore routine excision is not indicated [14]. However, Cullen et al. believe that surgical morbidity is higher for therapeutic resection than for prophylactic resection (12% vs 2%) [15]. Thirunavukarasu et al. recommended routine resection, stating that MD was associated with a high risk for ileal cancer [11]. In addition, carcinoid tumors appear to have a higher incidence in MD [16-17]. Some other teams suggested that surgical management should be decided on a case-by-case basis. In 2005, the Mayo Clinic completed a retrospective study of 1476 patients and identified the following risk factors for MD: age < 50 years old, male gender, length > 2 cm, and macroscopic abnormalities suggesting the presence of mucosal heterotopy. The combination of these four risk factors was associated with a 70% risk of complications, justifying prophylactic excision. The authors also recommended resection for all asymptomatic MD when at least one of these factors was present [6]. A risk score has been used by some to guide clinical decision-making [3,18]. Although promising, the current risk score calculator accounts for only four risk factors: male gender, patients <45 years of age, MD >2 cm, and the presence of a fibrous band. Long-term studies that correlate the presence of other comorbidities in these patients may help determine increased baseline risk for the transition from asymptomatic to symptomatic MD. Such studies would enable us to create a more refined MD risk score calculator to determine if surgery is warranted.

## Conclusions

Our case report challenges physicians to move away from the idea that MD is solely a neonatal disorder and to consider MD as a potential cause of GI pathology in the adult population, after appropriately excluding other, more common etiologies. Early identification and treatment lead to better outcomes for adults with potentially enigmatic GI bleeds caused by MD.

While widely accepted that symptomatic MD in the adult population be treated with surgical resection, prophylactic resection of incidentally discovered MD remains a topic of debate. It is our recommendation that management in this small population is considered on a case-by-case basis, through a multifactorial approach that includes the patient’s clinical status, lifelong risk of MD-related complications, anatomic features associated with the development of symptoms, and complications of an invasive procedure. We are of the opinion that a primary-care physician-guided risk-versus-benefit discussion be held to determine the best course of action, with immense focus paid to the aforementioned variables.
